# Maternal high fat intake affects the development and transcriptional profile of fetal intestine in late gestation using pig model

**DOI:** 10.1186/s12944-016-0261-0

**Published:** 2016-05-10

**Authors:** Lianqiang Che, Peilin Liu, Zhengguo Yang, Long Che, Liang Hu, Linlin Qin, Ru Wang, Zhengfeng Fang, Yan Lin, Shengyu Xu, Bin Feng, Jian Li, De Wu

**Affiliations:** Institute of Animal Nutrition, Sichuan Agricultural University, No.46, Xinkang Road, Ya’an, Sichuan 625014 People’s Republic of China; Key Laboratory for Animal Disease-Resistance Nutrition, Ministry of Education, No.46, Xinkang Road, Ya’an, Sichuan 625014 People’s Republic of China

**Keywords:** Maternal nutrition, Offspring, Immune, Cancer, DNA microarray

## Abstract

**Background:**

The objective of this study was to investigate the effects of maternal high fat intake on intestinal development and transcriptional profile.

**Methods:**

Eight gilts with similar age and body weight were randomly allocated into 2 groups receiving the control and high fat diets (HF diet) from d 30 to 90 of gestation, with 4 gilts each group and one gilt each pen. At d 90 of gestation, two fetuses each gilt were removed by cesarean section. Intestinal samples were collected for analysis of morphology, enzyme activities and transcriptional profile.

**Results:**

The results showed that feeding HF diet markedly increased the fetal weight and lactase activity, also tended to increase intestinal morphology. Porcine Oligo Microarray analysis indicated that feeding HF diet inhibited 64 % of genes (39 genes down-regulated while 22 genes up-regulated),which were related to immune response, cancer and metabolism, also markedly modified 33 signal pathways such as antigen processing and presentation, intestinal immune network for IgA production, Jak-STAT and TGF-ß signaling transductions, pathways in colorectal cancer and glycerolipid metabolism.

**Conclusion:**

Collectively, it could be concluded that maternal high fat intake was able to increase fetal weight and lactase activity, however, it altered the intestinal immune response, signal transduction and metabolism.

**Electronic supplementary material:**

The online version of this article (doi:10.1186/s12944-016-0261-0) contains supplementary material, which is available to authorized users.

## Background

Gastrointestinal tract (GIT), as an internal organ to digest nutrients and resist exogenous antigens, starts to develop at early gestation and mature rapidly in late gestation for extra-uterine life [1]. The functional maturation of GIT occurs in both pre- and postnatal period, which is largely influenced by maternal nutrition [2]. Maternal diet has been shown to affect the fetal development and organ function in mammalian animals [3]. Our recent study also suggests that maternal nutrition levels could affect the intestinal development and function, in which maternal over-nutrition would improve intestinal morphology, enzyme activities and gene expressions of nutrient transporters in newborn pigs [4]. However, it has been reported that maternal high-fat intake or –related obesity could impair gut barrier, enhance gene expression of pro-inflammatory cytokines in offspring intestine, thus predisposes offspring to inflammatory bowel disease [5, 6]. However, the underlying mechanism for the effects of maternal high fat intake on the intestinal development and function are limited. The current study was designed to investigate the effects of maternal high fat intake on fetal intestinal development and function by measuring parameters on morphology, enzyme activities and transcriptional profiles. Oligo Microarray was used to analyze the genomic response of fetal intestine to maternal high fat intake. Pigs were chosen as the experimental animal, because it is generally accepted to be closer to humans than other laboratory or domestic animals in terms of gastrointestinal anatomy, physiology, nutrition and microbiota [2, 7–9].

## Methods

The experimental procedure was approved by the University of Sichuan Agricultural Animal Care Advisory committee, and followed the current law of animal protection.

### Animals and diets

A total of 8 Meishan (MS) gilts (aged at 266 ± 15 d, initial body weight at 73 ± 4 kg) were used in this study. After inseminated with MS semen, eight gilts were randomly allocated to receive control diet (CON diet with 14 % Protein, 34.7 % Starch and 2.8 % Fat) and high fat diet (HF diet with 14 % Protein, 34.7 % Starch and 7.3 % Fat), respectively. The 4.5 % of soy oil was added into CON diet to formulate HF diet, as a result, HF diet contained digestive energy (DE) at 3.0 Mcal/kg, while CON diet contained DE at 2.6 Mcal/kg. According to the fatty acids contents of feed ingredients by NRC (2012), the contents of saturated, mono- and polyunsaturated fatty acids were 0.25 %, 0.48 %, 0.83 % in CON diet and 0.84 %, 1.78 %, 3.44 % in HF diet, respectively. The other nutrient levels were similar between 2 diets, meeting or exceeding nutrient requirements recommended by NRC (2012). All gilts were housed individually in stall (2.5 m length × 1.6 m width), receiving the same amount of diets at 2.0 kg from d 1 to 30 of gestation and 2.5 kg from d 30 to 90 of gestation, with free access to water. Environmental temperature was maintained at approximately 24 °C during the experiment.

### Sample collection

At d 90 of gestation, gilts were weighed (in average 128 kg at HF vs. 117 kg at CON group) and anaesthetized by intramuscularly injecting Zoletil 50 at the dose of 0.1 mg/kg (Virbac, France), then the uterus were removed from gilts. Two fetuses near the average fetal weight were collected each gilt. As the previous study, duodenal, jejunal and ileal samples (approximately 2 cm) were preserved in 4 % paraformaldehyde solution, then embedded in paraffin. Each tissue sample of duodenum, jejunum and ileum was used to prepare 5 slides, each slide had three sections (5 mm thickness), which were stained with eosin and haematoxylin, 20 well-oriented villi and crypts each section were measured for morphology (Optimus software version 6.5, Media Cybergenetics, North Reading, MA, USA), and villous height to crypt depth ratio (VCR) was calculated [10]. A section of duodenum, jejunum and ileum tissues were collected and snap-frozen in liquid nitrogen, then stored at −80 °C for analysis of enzyme activities, RNA microarray and gene expression.

### Enzyme activities

According to the previous study, the thawing samples of jejunum and ileum were weighed (approximately 2 g), then 9 times volume of 50 mM Tris–HCl buffer (pH 7 · 0) than the sample weight were added and homogenized for 40 s by homogenate machine (Homogenizer Power Gen 125™, ThermoFisher Scientific, MA, USA) and centrifuged at 3000 g for 10 min, the supernatant was collected and stored at −20 °C [11]. Total protein was extracted from the supernatant and protein concentration was determined by bicinchoninic acid protein assay with bovine serum albumin as the standard (Solarbio, Inc., Beijing, China). Activities of disaccharidase including maltase, sucrase and lactase were measured using commercial kits (Nanjing Jiancheng Bioengineering, Nanjing, China). The absorbance at 450 nm was determined with spectrophotometer (Beckman Coulter DU-800; Beckman Coulter, Inc., CA, USA). Activities of disaccharidase were presented as U/mg protein. One unit (U) was defined by 1 nmol of maltose, sucrose and lactose as a substrate for the enzymatic reaction, respectively.

### RNA extraction

The frozen ileum tissues were used for RNA extraction, 4 sections around luminal circle each tissue were collected and pooled for RNA extraction. Total RNA was extracted using Trizol reagent (Invitrogen, Carlsbad, CA, USA) and quantified using spectrophotometry based on absorbance at 260 nm, the RNA quality was monitored using Agilent 2100 Bioanalyzer (Agilent Technologies, Palo Alto, CA, USA). The equal amount of RNA from 2 fetus each gilt were pooled together.

### Porcine oligo microarray

As in our previous study, Agilent Porcine Oligo Microarray (4 × 44 K) containing more than 40,000 probes were used [12]. Cyanine-3 (Cy3)-labeled cRNA was prepared from 0.5 μg RNA using the One-Color Low RNA Input Linear Amplification PLUS kit (Agilent Technologies,Palo Alto, CA, USA) according to the manufacturer’s instructions, and followed by the RNeasy column purification (Qiagen, Valencia, CA, USA). Dye incorporation and cRNA yield were checked with the NanoDrop ND-1000 Spectrophotometer. Microarrays were hybridized at 65 °C for 17 h and washed with a Gene Expression Washing Buffer Kit (Agilent Technologies, Palo Alto, CA, USA). Slides were scanned with an Agilent microarray scanner.

### Microarray data collection and analysis

Microarray data were collected and analyzed using Agilent G2567AA Feature Extraction software, following Agilent’s direct labeling protocol. The quantile method was used to normalize the probe intensities across the whole set of arrays. Three criteria were used to determine statistically significant differential expression of intestinal genes between fetus from CON and HF gilts: 1) statistical significance: *P* value as determined by *t*-test < 0.05; 2) reliability: a spot quality flag P (“P,” a quality flag assigned by the software package); 3) relevance: a minimal fold change between the means of the 2 groups >1.5.

### Real-time PCR

In order to verify the microarray data, RNA samples used for porcine oligo microarray were applied to the quantitative real-time PCR (qPCR), which was performed in duplicate to amplify the target and reference genes, using one step SYBR Prime-Script™ RT-PCR kit II (Catalog no. DRR086A, Takara, Japan) by Real-Time PCR (ABI 7900HT, Applied Biosystems, CA, USA). The sequences of primers and length of products were shown at Table 1. The reaction mixture (10.0 μL) contained 5.6 μL of freshly pre-mixed one step SYBR Green Real-Time PCR Master mix and Prime Script™ Enzyme Mix, 0.8 μmol/L of the primers, and 100 ng of RNA template. The qPCR program was designed with one cycle of 42 °C for 5 min, one cycle of 95 °C for 10 s, 40 cycles of 95 °C for 5 s and 60 °C for 34 s, followed by the dissociation step at 95 °C for 15 s, 60 °C for 60 s and 95 °C for 15 s. At the end of amplification, melting curve analysis was performed to identify amplification specificity. Amplification of ß-actin was used to normalize gene expression through the double standard curves method [11].

**Table 1:** Primer sequences of genes selected for analysis by real-time RT-PCR

Genes	GenBank accession	Primer sequence (5′ ~ 3′)	Product length (bp)	Tm (°C)
HSPA1L	NM_001123128.1	F:CGCTTTGACCTGACTGGAAT	120	60
		R:CTTGCCTGTGCTCTTGTCC		
CD8A	NM_001001907.1	F:GCTGGACACCCGTTACATCT	100	60
		R:CGAGCAGAAATAGTAGCCTTGG		
CD40	NM_214194.1	F:GGTTCGTCTGCCTCTGAAGT	104	60
		R:GGCTGTTTGTTGGGTATTGG		
PSTPIP1	NM_001244186.1	F:CTCCTTTGACTCCCTGAAGC	114	60
		R:TTCTGCCTCTCTCGGAACTC		
SLA-DQA1	NM_001114062.2	F:TGGACCTGGAGAAGAAGGAG	132	60
		R:TGGAGCGTTTAGTCACGATG		
STAT2	NM_213889.1	F:TCCCAAATCACAAGGTTTCC	109	60
		R:CAGATAGCCGAAGTCCCAAA		
GK	NM_001143708.1	F:GCAGGTAGATGGAGGGATGA	107	60
		R:CCAGGGCAGTTGTTTCAGG		
BMP7	NM_001105290.1	F:TCCAGGGCAAGCACAACT	172	60
		R:TCGGTGAGGAAGTGGCTATC		
PIK3R5	NM_213851.1	F:CTGTCATTCCCTCCTTCCAA	117	60
		R:GCCACCCTCCTCTTACTCTG		
SLA-DRB1	NM_001113695.1	F:TCTGCTCTTTGTTGCTGTGG	120	60
		R:GGATGCTTGCTTGGAGTGTC		
THY1	XM_005667396.1	F:GGCATCGCTCTCTTGCTAAC	125	60
		R:GGCAGGTTGGTGGTATTCTC		
TGFB1	NM_214015.1	F:AAGCGGCAACCAAATCTATG	113	60
		R:CCCGAGAGAGCAATACAGGT		
SLA-1	NM_001097431.1	F:GTCAAGGAAACCGCACAGAT	113	60
		R:CCCAAGTAGCAGCCAAACAT		
CD74	NM_213774.1	F:ATGGACGGTGTGAACTGGA	100	60
		R:GAACCTCAAAGGGTGTCTCCT		
SOD2	XM_005659113.1	F:CCTTCACTTTGCCTCTTGGT	127	60
		R:CACCGTTAGGGCTCAGATTT		
ACTA2	XM_005671254.1	F:GTCCACCTTCCAGCAAATGT	105	60
		R:AGACAGCGAGCAGGGTAAGT		
SULT1E1	NM_213992.1	F:TGAAGTCTCATCTGCCACCT	101	60
		R:AGAAACGACCACATCCTTGG		
β-actin	DQ845171.1	F:GGCGCCCAGCACGAT	66	60
		R:CCGATCCACACGGAGTACTTG		

*HSPA1L* heat shock 70 kDa protein 1-like, *CD8A* CD8a molecule (CD8A), *CD40* CD40 molecule, TNF receptor superfamily member 5; *SLA-DQA1* MHC class II histocompatibility antigen SLA-DQA, *PSTPIP1* proline-serine-threonine phosphatase interacting protein 1, *STAT2* signal transducer and activator of transcription 2, *GK* glycerol kinase, *BMP7* bone morphogenetic protein 7, *PIK3R5* phosphoinositide-3-kinase, regulatory subunit 5, *SLA-DRB1* MHC class II histocompatibility antigen SLA-DRB1, *THY1* Thy-1 cell surface antigen, *TGFB1* transforming growth factor, beta 1, *SLA-1* MHC class I antigen 1, *CD74* CD74 molecule, major histocompatibility complex, class II invariant chain, *SOD2* superoxide dismutase 2, mitochondrial, *ACTA2* actin, alpha 2, smooth muscle, aorta, *SULT1E1* sulfotransferase family 1E, estrogen-preferring, member 1

### Statistical analysis

The detected data by samples from two fetuses each gilt were averaged and taken as one independent data involving into statistical analysis model. In addition to Oligo Microarray and qPCR data, all other data on growth performance, intestinal morphology and enzyme activities were analyzed via the t Student’s *t* test for a completely randomized design using SAS (SAS, Cary, NC). Results were expressed as the mean ± SD. Differences were considered to be significant when *P* <0.05, while a tendency was considered when 0.05 < *P* < 0.10.

## Results

### Growth performance

Feeding HF diet markedly increased the fetal weight (in average 585 g vs.508 g, *P* < 0.05) at d 90 of gestation.

### Morphology and enzyme activities

**Fig. 1:**
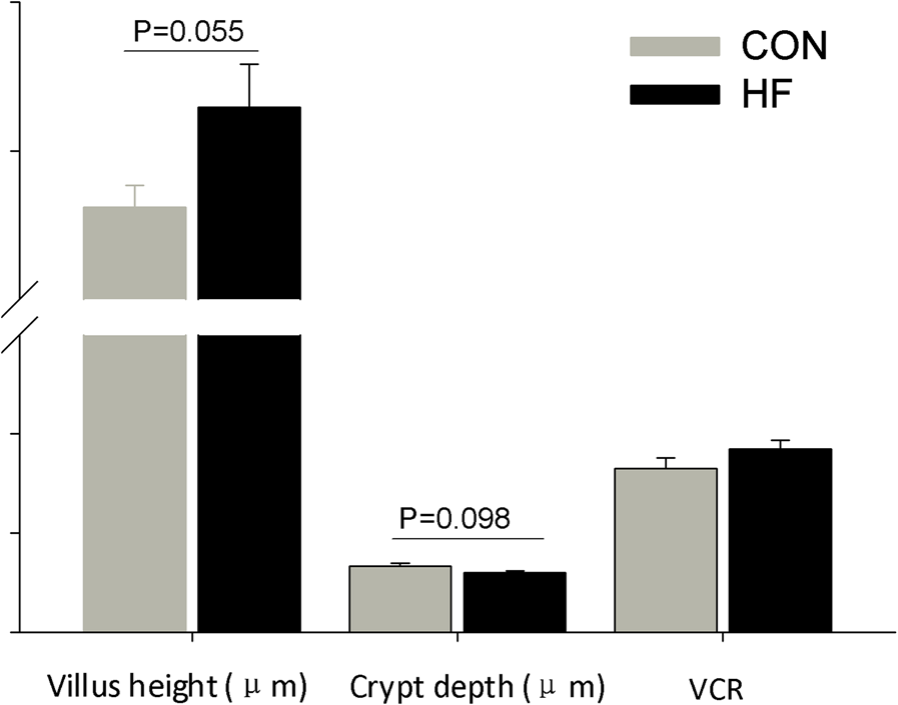
Effect of maternal high fat intake on the intestinal morphology of fetus (*n* = 4)

**Fig. 2:**
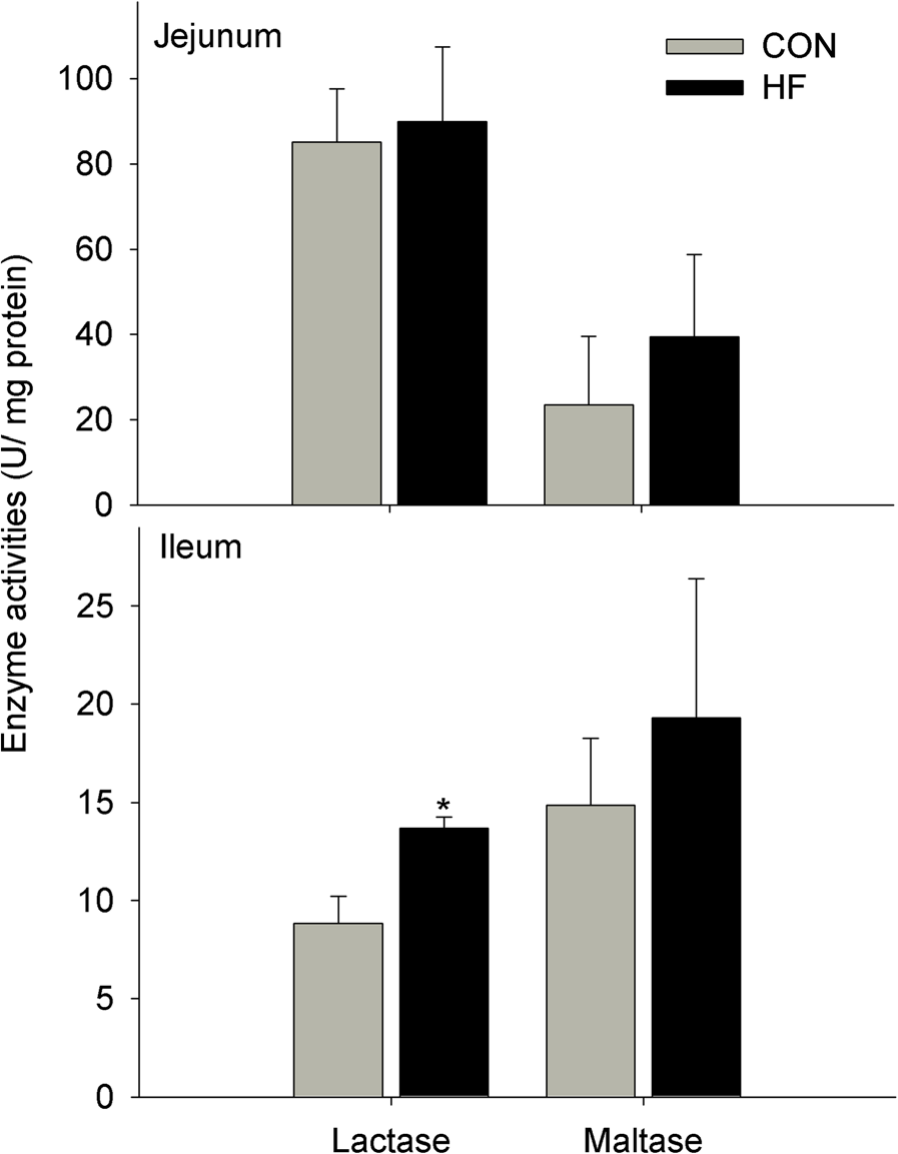
Effects of maternal high fat intake on digestive enzyme activities of fetal intestine (*n* = 4). The symbol “*” in figure represents there was significant difference at 5 % level (*P* < 0.05)

Feeding HF diet tended to increase intestinal villous height (*P* = 0.055), but decrease crypt depth (*P* = 0.098) of fetus (Fig. 1). Meanwhile, the lactase activity was markedly increased (+55 %, *P* < 0.05) by feeding HF diet relative to CON diet, whereas the maltase activity did not markedly differ between groups (Fig. 2), and sucrase activity could not be detected in fetal intestine. Gene expression of digestive enzymes were not markedly differ between two groups (Additional file 1).

### Differentially expressed genes in fetal intestine

**Fig. 3:**
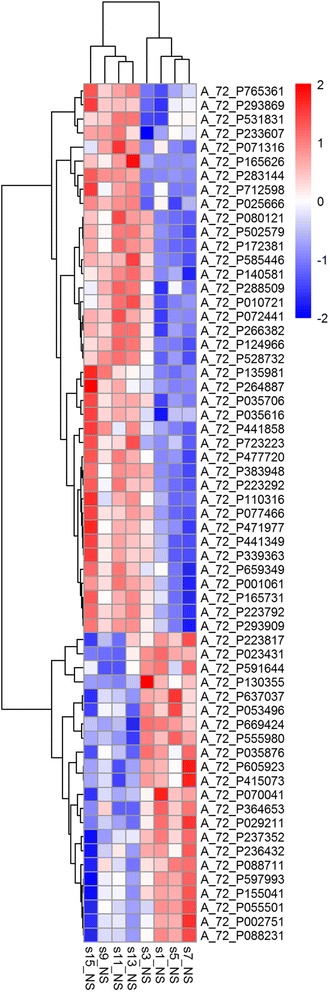
Heatmap of the 61 differentially expressed genes. The HF diet: s1_NS, s3-NS, s5-NS, s7-NS; The CON diet: s9_NS, s11-NS, s13-NS, s15-NS

A total of 61 genes were differentially expressed (at least 1.5 fold change, *P* < 0.05), and 39 genes were down-regulated while 22 genes were up-regulated (Table 2, Fig. 3). The changes in mRNA expression detected by porcine oligo microarrays were further validated by qRT-PCR (Table 3). Given their participation in crucial biological process and modulating signal pathways on immune response, cancer and metabolism, these genes were chosen for Real-Time PCR analysis.

**Table 2:** Maternal high fat intake markedly regulated intestinal gene expressions related to immune response, signal transduction, cancer and metabolism

Gene Symbol^a^	Gene name	Fold change^b^	*P* value
CCR7	chemokine (C-C motif) receptor 7	−2.94	0.023
HSPA1L	heat shock 70 kDa protein 1-like	−2.50	0.016
CD8A	CD8a molecule (CD8A)	−2.44	0.035
CD3E	CD3e molecule, epsilon (CD3-TCR complex) (CD3E)	−2.27	0.033
STK17B	serine/threonine kinase 17b	−2.00	0.026
CD40	CD40 molecule, TNF receptor superfamily member 5	−2.00	0.011
CD2	CD2 molecule	−1.89	0.026
SLA-DQA1	MHC class II histocompatibility antigen SLA-DQA	−1.85	0.002
PSTPIP1	proline-serine-threonine phosphatase interacting protein 1	−1.85	0.007
SLAMF6	SLAM family member 6	−1.82	0.046
TP53INP1	tumor protein p53 inducible nuclear protein 1	−1.82	0.000
FAM78A	family with sequence similarity 78, member A	−1.79	0.015
BCL2A1	BCL2-related protein A1	−1.79	0.023
ARHGAP25	Rho GTPase activating protein 25	−1.75	0.011
CD1.1	CD1 antigen	−1.72	0.007
STAT2	signal transducer and activator of transcription 2	−1.69	0.042
ARHGAP30	Rho GTPase activating protein 30	−1.69	0.036
BCL2A1	BCL2-related protein A1	−1.69	0.040
IL10RB	interleukin 10 receptor, beta	−1.67	0.013
GK	glycerol kinase	−1.64	0.041
LTB	mRNA, clone:MLN010057G03, expressed in mesenteric lymph nodes	−1.64	0.031
LCP1	lymphocyte cytosolic protein 1 (L-plastin)	−1.61	0.014
PGM1	phosphoglucomutase 1	−1.61	0.045
NRROS	negative regulator of reactive oxygen species	−1.59	0.049
CYTH4	cytohesin 4	−1.59	0.039
BMP7	bone morphogenetic protein 7	−1.59	0.024
PIK3R5	phosphoinositide-3-kinase, regulatory subunit 5	−1.56	0.009
RGS14	regulator of G-protein signaling 14	−1.54	0.049
GLRX	glutaredoxin (thioltransferase)	−1.54	0.025
SLA-DRB1	MHC class II histocompatibility antigen SLA-DRB1	−1.52	0.028
LPAR2	lysophosphatidic acid receptor 2	−1.52	0.016
THY1	Thy-1 cell surface antigen	−1.52	0.028
TGFB1	transforming growth factor, beta 1	−1.52	0.022
BAZ1A	bromodomain adjacent to zinc finger domain, 1A	−1.52	0.024
CCDC69	coiled-coil domain containing 69	−1.49	0.048
LRRK2	leucine-rich repeat kinase 2	−1.49	0.022
SLA-1	MHC class I antigen 1	−1.49	0.018
CD74	CD74 molecule, major histocompatibility complex, class II invariant chain	−1.49	0.038
SOD2	superoxide dismutase 2, mitochondrial	1.51	0.004
ILF2	interleukin enhancer binding factor 2	1.51	0.021
CYP39A1	cytochrome P450, family 39, subfamily A, polypeptide 1	1.52	0.043
JPH4	junctophilin 4	1.52	0.026
ATCAY	ataxia, cerebellar, Cayman type	1.53	0.008
MATN2	mRNA, clone:OVR010041A03, expressed in ovary	1.53	0.016
CRMP1	Uncharacterized protein	1.54	0.039
RTDR1	mRNA, clone:UTR010010H08, expressed in uterus.	1.55	0.001
SPARCL1	SPARC-like 1 (hevin)	1.56	0.035
MATN2	mRNA, clone:OVR010041A03, expressed in ovary	1.56	0.019
CCN2	connective tissue growth factor	1.57	0.042
TUSC3	mRNA, clone: HTMT10103A12, expressed in hypothalamus	1.58	0.009
ID4	inhibitor of DNA binding 4, dominant negative helix-loop-helix protein	1.58	0.024
SPARC	secreted protein, acidic, cysteine-rich (osteonectin)	1.60	0.035
MEP1A	meprin A, alpha (PABA peptide hydrolase)	1.63	0.018
ARL10	ADP-ribosylation factor-like 10	1.64	0.036
STMN2	stathmin-like 2	1.64	0.039
ACTA2	actin, alpha 2, smooth muscle, aorta	1.66	0.016
SHISA2	shisa family member 2	1.76	0.029
UCHL1	ubiquitin carboxyl-terminal esterase L1 (ubiquitin thiolesterase)	1.79	0.014
OCRL	oculocerebrorenal syndrome of Lowe	2.01	0.008
SULT1E1	sulfotransferase family 1E, estrogen-preferring, member 1	2.59	0.013

^a^Genes were selected from the Kyoto Encyclopedia of Genes and Genomes pathways related to intestinal immune response, signal transduction, cancer and metabolism (http://www.genome.jp/kegg/pathway.html)

^b^The fold change was based on the ratio of HF group to CON group, *n* = 4 subpools/group

**Table 3:** Differentially expressed genes in fetal intestine by maternal high fat intake and validated by qPCR

		Fold change^b^	
Gene symbol^a^	cDNA Microarray	qPCR	*P* value
ACTA2	1.66	1.10	0.246
SULT1E1	2.59	1.88	0.002
SOD2	1.51	1.75	0.036
BMP7	−1.59	−1.09	0.722
CD40	−2.00	−1.74	0.116
CD74	−1.49	−1.36	0.029
CD8A	−2.44	−1.85	0.049
GK	−1.64	−1.33	0.041
HSPAIL	−2.50	−1.60	0.027
PIK3R5	−1.56	−1.07	0.644
PSTPIP1	−1.85	−1.48	0.083
SLA-1	−1.49	−1.45	0.097
SLA-DQA1	−1.85	−1.79	0.003
SLA-DRB1	−1.52	−1.33	0.136
STAT2	−1.69	−1.18	0.125
TGF-β	−1.52	−1.19	0.296
THY1	−1.52	−1.29	0.118

^a^Genes were selected on the basis of their crucial role on regulating intestinal immune response (i.e.SLA-DRB1,SLA-DQA,HSPA1L,CD74,CD40), colorectal cancer (i.e.TGF-β,PIK3R5), signal transduction (i.e. PSTPIP1,BMP7,STAT2) and metabolism (i.e.GK, SULT1E1). These genes by DNA microarray were all significantly regulated (*P* < 0.05, at least 1.5 fold change)

^b^The fold change was based on the ratio of HF group to CON group, *n* = 4 subpools/group

### Analysis of gene ontology and signal pathway

**Fig. 4:**
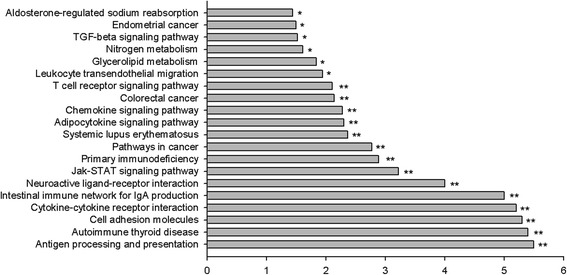
Signal pathway enrichment analysis of fetal intestine by HF diet relative to CON diet (*n* = 4 subpools/group). The pathway terms were according to the down-regulated genes for certain biological processes, enriched categories are those identified as significantly enriched after multiple testing. * *P* < 0.05, ** *P* < 0.01. The value by horizontal axis resulted from negative value of Log (enrichment test *P* value, base 10)

The differentially expressed genes were clustered according to their biological process ontology by Gene Ontology (GO) analysis from the SBS analysis system (http://www.shanghaibiotech.com/). A large number of these genes were associated with antigen processing and presentation [i.e. D74, CD8A, SLA-DOB, SLA-DRB1, SLA-DQA, HSPA1L], intestinal immune network for IgA production [i.e. CD40, IL6, TGFβ1], Jak-STAT signaling pathway [i.e. IL6, STAT2 and PIK3R5], TGF-ß signaling pathway [i.e. TGF-β and PIK3R5], pathways in cancer [i.e. LEF1, PIK3R5, NOS2] and glycerolipid metabolism [i.e. GK, PNLIPRP1] et al. (Table 2, Fig. 4).

Consequently, maternal HF intake markedly modified 33 signal pathways (*P* < 0.01) (Table 4), which were mainly involved in immune response (i.e. antigen processing and presentation, intestinal immune network for IgA production, primary immunodeficiency), signaling transduction (i.e. TGF-ß signaling pathway, chemokine signaling pathway), cancer (i.e. colorectal cancer, pathways in cancer), metabolism (i.e. glycerolipid metabolism, nitrogen metabolism), signaling molecules and interaction (i.e. cytokine-cytokine receptor interaction, cell adhesion molecules, neuroactive ligand-receptor interaction).

**Table 4:** The markedly modified signal pathways in fetal intestine of gilts fed HF diet

Name	Hits ^a^	Total ^b^	Percent	Enrichment test
				*p* value
Allograft rejection	6	34	17.65 %	0.000
Antigen processing and presentation	8	64	12.50 %	0.000
Autoimmune thyroid disease	6	45	13.33 %	0.000
Cell adhesion molecules	8	71	11.27 %	0.000
Cytokine-cytokine receptor interaction	10	142	7.04 %	0.000
Hematopoietic cell lineage	7	63	11.11 %	0.000
Intestinal immune network for IgA production	7	48	14.58 %	0.000
Leishmania infection	8	63	12.70 %	0.000
Viral myocarditis	9	46	19.57 %	0.000
Graft-versus-host disease	6	57	10.53 %	1E-04
Neuroactive ligand-receptor interaction	10	174	5.75 %	1E-04
Type I diabetes mellitus	5	40	12.50 %	2E-04
Asthma	5	50	10.00 %	5E-04
Jak-STAT signaling pathway	6	82	7.32 %	6E-04
Primary immunodeficiency	4	37	10.81 %	0.0013
Pathways in cancer	7	140	5.00 %	0.0017
Hypertrophic cardiomyopathy	4	43	9.30 %	0.0022
Systemic lupus erythematosus	5	86	5.81 %	0.0043
Adipocytokine signaling pathway	4	55	7.27 %	0.005
Chemokine signaling pathway	5	90	5.56 %	0.0052
Colorectal cancer	3	31	9.68 %	0.0072
Fc gamma R-mediated phagocytosis	3	32	9.38 %	0.0078
T cell receptor signaling pathway	4	63	6.35 %	0.0078
Leukocyte transendothelial migration	4	71	5.63 %	0.0115
Acute myeloid leukemia	3	39	7.69 %	0.0129
Dilated cardiomyopathy	3	40	7.50 %	0.0137
Glycerolipid metabolism	3	41	7.32 %	0.0146
Arrhythmogenic right ventricular cardiomyopathy	3	47	6.38 %	0.0205
Nitrogen metabolism	2	19	10.53 %	0.0246
TGF-beta signaling pathway	3	55	5.45 %	0.0302
Endometrial cancer	2	22	9.09 %	0.0316
Aldosterone-regulated sodium reabsorption	2	24	8.33 %	0.0366
Type II diabetes mellitus	2	24	8.33 %	0.0366

^a^Hits mean the number of differential expressed genes within the particular GO term

^b^Total: the total number of genes within the particular GO term

## Discussion

Some studies have indicated that maternal nutrition would affect the intestinal development and function of offspring [4, 13–15].

In this study, maternal high fat intake increased intestinal villous height and lactase activity, which is similar as our recent study that maternal over-nutrition markedly increased birth weight, accordingly intestinal morphology as well as lactase activity [4]. It may be rational that the heavier birth weight needs higher lactase activity in preparation for better degradation of lactose, which is a crucial energy source in neonatal period [16]. However, a recent study indicated that maternal high fat intake would induce intestinal inflammation and poor gut barrier function in the offspring of mice [5]. In this study, porcine oligo miacro array analysis was used to determine the genomic response of intestine to maternal high fat intake, in an attempt to reveal the potential mechanism. According to the strict selection criteria, we found a total of 61 genes were differentially regulated and 64 % of them (39 genes) was down-regulated by HF diet. With the bioinformatics analysis, these down-regulated genes were mainly involved in process of immune response, signaling transduction, pathways in cancer and metabolism, suggesting the inhibitory effects of maternal high fat intake on certain biological events. The maternal diet fat composition could change the maternal-to-fetal fatty acid transfer and intestinal membrane n-6 and n-3 fatty acids composition of newborns, thus altering intestinal function [13]. In this study, therefore, it is rational that the addition of soy oil in maternal diet would induce alterations in intestinal physiology of fetus. Obviously, antigen processing and presentation in intestine could be inhibited by feeding HF diet, as indicated by the markedly decreasing gene expressions (i.e. SLA-1, SLA -DRB1, SLA-DQA1, CD74 and CD8, 1.5 ~ 2.5 fold reduction). Particularly, SLA-1, SLA -DRB1 and SLA-DQA1 are belonged to the highly polymorphic swine leucocyte antigen genes, which determine the immune response to disease and vaccine [17]. Among them, SLA-1 could interact with natural killer cells to prevent cytotoxicity [18], while SLA-DRB1 and -DQA1 mainly present exogenous peptides for T cells [18, 19]. Previous studies have shown that maternal high fat intake impaired intestinal barrier and immune system through altering immune cell homeostasis, such as the number of T cells and macrophages [13]. Furthermore, intestinal immune network for IgA production may be impaired by HF diet, as shown by the decreasing gene expression of CD40, IL-6 and TGF-ß. These genes are required for B cells proliferation and differentiation in Peyer’s patches, their down-regulation would reduce the homing of T cells and IgA^+^ plasma cells to the intestine, thus impair the immune homeostasis of intestine [20, 21].

Several signal transduction pathways related to inflammatory and immune response were affected by maternal high fat intake. For example, the TGF-β signaling pathway was affected by HF diet, as indicated by the decreasing gene expression of TGF-β and Bmp7 (approximately 1.6 fold reduction). TGF-β is a multifunctional factor regulating cell growth, adhesion and differentiation [22, 23], also exerting anti-inflammatory effects by inhibiting NF-κB expression in the intestinal epithelium [24]. The oral administration of TGF-β has been shown to decrease severity and incidence of necrotizing enterocolitis in neonatal rat necrotizing enterocolitis model [24]. In addition, feeding HF diet affected intestinal Jak-STAT signaling pathway, as shown by the decreasing gene expression of IL6, STAT2 and PIK3R5. The Jak-STAT signaling pathway is required for T cell differentiation, B cell maturation and secretion of sIgA [25], these down-regulated genes by HF diet may induce the abnormal intestinal innate immune response. Similarly, previous study demonstrated that maternal high protein diet would decrease liver mass, associated with altering gene expressions mapping to Jak-STAT signaling pathway in mouse offspring [26].

Furthermore, the lower expressions of TGF-β and PIK3R5 genes by HF diet may affect the progression of colorectal cancer. TGF-β1/Smads signaling pathway was demonstrated to mediate epithelial-to-mesenchymal transition, associated with the progression of colorectal cancer [27]. Mutation of PIK3R5 and other genes (i.e. PRKCZ, PTEN, RHEB and RPS6KB1) have altered PI3K signaling pathway, which is the central pathway for both colorectal and breast cancers [28]. Recent studies also indicated that maternal high fat diet would modify the susceptibility to breast cancer [29, 30], meanwhile it is dependent on fat or oil sources [31–33].

In this study, moreover, the markedly reduced glycerol kinase by feeding HF diet suggests the intestinal metabolism was altered. Glycerol kinase is required to release glycerol from glycerol-3-phosphate and dihydroxyacetone, and intestinal glycerol could produce 20 ~ 25 % of total endogenous glucose under insulinopenia, suggesting the important role of glycerol in intestinal metabolism [34]. Although most of genes were markedly down-regulated by HF diet, some of genes (SOD2, CYP39A1, CCN2, SPARC et al.) were up-regulated. Particularly, SOD2, as an anti-oxidative enzyme in living cells, was highly expressed (1.75 fold change, *P* = 0.04). Likewise, a recent study demonstrated that maternal high energy intake increased the expression of SOD in offspring ileum [5]. Previous study indicated that the increasing SOD gene is not necessarily associated with a better antioxidant capability, for example, the inflamed intestinal mucosa has been shown to contain higher SOD protein compared with normal tissues [35]. In addition, we found that feeding HF diet markedly increased gene expression of SULT1E by both DNA Microarray and RT-PCR analysis. SULT1E, as an estrogen-preferring drug metabolizing enzyme, its highly expression may be an compensatory response to high circulating estrogen, which occurs in dams fed high fat diet [36]. It has been shown that the estrogen deletion by SULT1E over-expression is associated with the risk of developing different types of cancers [28, 37].

## Conclusion

In summary, maternal high fat intake was able to increase fetal and intestinal weights as well as lactase activity, however, it altered the intestinal immune response, signal transduction and metabolism.

## Additional file

Additional file 1: Effect of maternal high fat intake on gene expression of digestive enzymes in fetal intestine. (DOCX 34 kb)
