# Supplementary material for: Maternal high fat intake affects the development and transcriptional profile of fetal intestine in late gestation using pig model
Source: Lipids Health Dis. 2016 May 10;15:90. doi: 10.1186/s12944-016-0261-0 (PMC4862081; doi:10.1186/s12944-016-0261-0)
Supplement: Additional file 1: — Effect of maternal high fat intake on gene expression of digestive enzymes in fetal intestine. (DOCX 34 kb) [file 12944_2016_261_MOESM1_ESM.docx]

LPH, lactase-phlorizin hydrolase; MGA, maltase-glucoamylase; SUC, sucrase-isomaltase
